# Prognostic impact of cachexia by multi‐assessment in older adults with heart failure: FRAGILE‐HF cohort study

**DOI:** 10.1002/jcsm.13291

**Published:** 2023-07-11

**Authors:** Emi Maekawa, Takumi Noda, Daichi Maeda, Masashi Yamashita, Shota Uchida, Nobuaki Hamazaki, Kohei Nozaki, Hiroshi Saito, Kazuya Saito, Yuki Ogasahara, Masaaki Konishi, Takeshi Kitai, Kentaro Iwata, Kentaro Jujo, Hiroshi Wada, Takatoshi Kasai, Hirofumi Nagamatsu, Tetsuya Ozawa, Katsuya Izawa, Shuhei Yamamoto, Naoki Aizawa, Ryusuke Yonezawa, Kazuhiro Oka, Junya Ako, Shin‐ichi Momomura, Nobuyuki Kagiyama, Yuya Matsue, Kentaro Kamiya

**Affiliations:** ^1^ Department of Cardiovascular Medicine Kitasato University School of Medicine Sagamihara Japan; ^2^ Department of Rehabilitation Sciences Kitasato University Graduate School of Medical Sciences Sagamihara Japan; ^3^ Department of Cardiovascular Biology and Medicine Juntendo University Graduate School of Medicine Tokyo Japan; ^4^ Department of Rehabilitation Kitasato University Hospital Sagamihara Japan; ^5^ Department of Rehabilitation Kameda Medical Center Kamogawa Japan; ^6^ Department of Rehabilitation The Sakakibara Heart Institute of Okayama Okayama Japan; ^7^ Department of Nursing The Sakakibara Heart Institute of Okayama Okayama Japan; ^8^ Division of Cardiology Yokohama City University Medical Center Yokohama Japan; ^9^ Department of Cardiovascular Medicine National Cerebral and Cardiovascular Center Suita Japan; ^10^ Department of Rehabilitation Kobe City Medical Center General Hospital Kobe Japan; ^11^ Department of Cardiology Nishiarai Heart Center Hospital Tokyo Japan; ^12^ Department of Cardiovascular Medicine, Saitama Medical Center Jichi Medical University Shimotsuke Japan; ^13^ Department of Cardiovascular Respiratory Sleep Medicine Juntendo University Graduate School of Medicine Tokyo Japan; ^14^ Department of Cardiology Tokai University School of Medicine Isehara Japan; ^15^ Department of Rehabilitation Odawara Municipal Hospital Odawara Japan; ^16^ Department of Rehabilitation Matsui Heart Clinic Saitama Japan; ^17^ Department of Rehabilitation Shinshu University Hospital Matsumoto Japan; ^18^ Department of Cardiovascular Medicine, Nephrology and Neurology University of the Ryukyus Nishihara Japan; ^19^ Department of Rehabilitation Kitasato University Medical Center Kitamoto Japan; ^20^ Department of Rehabilitation Saitama Citizens Medical Center Saitama Japan; ^21^ Saitama Citizens Medical Center Saitama Japan; ^22^ Department of Cardiovascular Biology and Medicine Juntendo University Faculty of Medicine Tokyo Japan; ^23^ Department of Cardiology The Sakakibara Heart Institute of Okayama Okayama Japan; ^24^ Department of Digital Health and Telemedicine R&D Juntendo University Tokyo Japan; ^25^ Department of Rehabilitation, School of Allied Health Sciences Kitasato University Sagamihara Japan

**Keywords:** cachexia, heart failure, older adults, prognosis

## Abstract

**Background:**

Cachexia substantially impacts the prognosis of patients with heart failure (HF); however, there is no standard method for cachexia diagnosis. This study aimed to investigate the association of Evans's criteria, consisting of multiple assessments, with the prognosis of HF in older adults.

**Methods:**

This study is a secondary analysis of the data from the FRAGILE‐HF study, a prospective multicentre cohort study that enrolled consecutive hospitalized patients aged ≥65 years with HF. Patients were divided into two groups: the cachexia and non‐cachexia groups. Cachexia was defined according to Evans's criteria by assessing weight loss, muscle weakness, fatigue, anorexia, a decreased fat‐free mass index and an abnormal biochemical profile. The primary outcome was all‐cause mortality, as assessed in the survival analysis.

**Results:**

Cachexia was present in 35.5% of the 1306 enrolled patients (median age [inter‐quartile range], 81 [74–86] years; 57.0% male); 59.6%, 73.2%, 15.6%, 71.0%, 44.9% and 64.6% had weight loss, decreased muscle strength, a low fat‐free mass index, abnormal biochemistry, anorexia and fatigue, respectively. All‐cause mortality occurred in 270 patients (21.0%) over 2 years. The cachexia group (hazard ratio [HR], 1.494; 95% confidence interval [CI], 1.173–1.903; *P* = 0.001) had a higher mortality risk than the non‐cachexia group after adjusting for the severity of HF. Cardiovascular and non‐cardiovascular deaths occurred in 148 (11.3%) and 122 patients (9.3%), respectively. The adjusted HRs for cachexia in cardiovascular mortality and non‐cardiovascular mortality were 1.456 (95% CI, 1.048–2.023; *P* = 0.025) and 1.561 (95% CI, 1.086–2.243; *P* = 0.017), respectively. Among the cachexia diagnostic criteria, decreased muscle strength (HR, 1.514; 95% CI, 1.095–2.093; *P* = 0.012) and low fat‐free mass index (HR, 1.424; 95% CI, 1.052–1.926; *P* = 0.022) were significantly associated with high all‐cause mortality, but there was no significant association between weight loss alone (HR, 1.147; 95% CI, 0.895–1.471; *P* = 0.277) and all‐cause mortality.

**Conclusions:**

Cachexia evaluated by multi‐assessment was present in one third of older adults with HF and was associated with a worse prognosis. A multimodal assessment of cachexia may be helpful for risk stratification in older patients with HF.

## Introduction

In developed countries with ageing populations, including Japan, the number of patients with heart failure (HF) has increased.^1^ In addition to increased mortality, HF causes various problems, such as a decreased quality of life (QOL) and an increased medical burden.^2^ Moreover, HF is known to cause weight loss, loss of muscle mass, chronic inflammation, malnutrition and exhaustion, which induce cachexia.^3^, ^4^

Cachexia, defined as involuntary weight loss, occurs in patients with various chronic diseases involving HF (such as cancer and chronic obstructive pulmonary disease patients)^5^ and is a vital independent risk factor for mortality.^6^, ^7^ Cachexia differs from loss of muscle mass due to starvation or ageing in that it is a complex underlying disease.^8^ It is a metabolic syndrome characterized by several overlapping problems (including muscle loss with or without loss of fat mass, anorexia, fatigue and inflammation).^5^, ^9^

Several studies have examined the frequency and prognosis of cachexia in patients with HF; however, most of these studies defined cachexia solely in terms of weight loss.^10^, ^11^, ^12^, ^13^ The exact frequency and prognostic impact of cachexia in patients with HF is unknown; however, there should be a comprehensive assessment of cachexia that includes muscle mass and strength (the weight of patients with HF fluctuates with the disease state and treatment process). Assessment of cachexia by comprehensive criteria may be helpful for detailed risk stratification in patients with HF, and patients with cachexia are predicted to have poor prognosis than those with weight loss alone.

This study aimed to examine the prevalence and prognostic impact of cachexia as defined by Evans's criteria,^5^ which is recommended for the comprehensive assessment of cachexia in older adults with HF, based on data from the prevalence and prognostic value of physical and social frailty in geriatric patients hospitalized for HF (FRAGILE‐HF) cohort study.

## Methods

### Study design and patient population

This is a secondary analysis of the FRAGILE‐HF study, a prospective, multicentre, observational study conducted at 15 hospitals in Japan.^14^ Briefly, we assessed eligibility for all consecutive patients aged ≥65 years who were first admitted due to HF decompensation between September 2016 and March 2018 and who were ambulatory at discharge. The Framingham criteria were used to diagnose HF decompensation.^15^ Patients with a history heart transplantation or left ventricular assist device implantation, patients on chronic peritoneal dialysis or haemodialysis, patients with acute myocarditis, B‐type natriuretic peptide (BNP) levels <100 pg/mL or N‐terminal pro‐BNP levels <300 pg/mL on admission, and patients for whom data were not available were excluded from the study. The study protocol was conducted in accordance with the tenets of the Declaration of Helsinki and was approved by the ethics committee of each participating hospital. Because this was an observational study that did not entail invasive procedures or interventions, written informed consent was not required under the Ethical Guidelines for Medical and Health Research Involving Human Subjects, per the Japanese Ministry of Health, Labour and Welfare. All participants were free to withdraw from the study at any time, and study information, including study objectives, inclusion and exclusion criteria, and names of participating institutions, was posted on the University hospital Medical Information Network (UMIN‐CTR, unique identifier: UMIN000023929) prior to patient enrolment.

### Data collection and cachexia assessment

Prior to discharge, all hemodynamically stable patients underwent baseline physical examination, blood sampling and echocardiography and were administered with medication. The Japanese Society of Nephrology formula was used to extrapolate the estimated glomerular filtration rate (eGFR).^16^

Cachexia status was assessed per the definition from Evans et al.,^5^ which is weight loss characterized by decreased muscle strength, fatigue, anorexia, a low fat‐free mass index and abnormal biochemistry (*Table* *S1*). Each of the five characteristics was defined as follows: (1) weight loss of at least 5% of the previous average weight within 12 months and/or a body mass index (BMI) <18.5 kg/m^2^. Because recalled past weight has been strongly correlated with measured weight,^17^, ^18^ weight loss was assessed using current weight and medical history interviews of recalled weight 1 year prior. We used BMI < 18.5 kg/m^2^ as a cutoff for weight loss^19^ to account for differences in body size between Asian and Western people. (2) Three or more of the following conditions were present: muscle weakness, fatigue, anorexia, a decreased fat‐free mass index and an abnormal biochemical profile (increased C‐reactive protein [CRP] >5.0 mg/L, decreased haemoglobin [Hb] <12 g/dL and low serum albumin <3.2 g/dL).

As measured using a dynamometer, handgrip strength was used to define muscle weakness; participants sat with their elbow joint flexed at 90° and performed the test, alternating between their right and left hands. The maximum reading from the two trials using both hands was expressed in kg. A handgrip strength of <28 kg for men and <18 kg for women was defined as muscle weakness according to the Asian Working Group for Sarcopenia 2019.^20^ Fatigue, defined as physical and/or mental weariness, and decreased food intake/anorexia were assessed qualitatively using questionnaires and interviews at the time of hospital discharge. The fat‐free mass index was assessed from the mid‐upper arm muscle circumference (MUAMC), and based on the Japanese Anthropometric Reference Data 2001,^21^ an MUAMC < 10th percentile for age and sex was defined as a decrease in MUAMC. MUAMC was calculated from the mid‐upper arm circumference (MUAC) and the triceps skinfold thickness (TSF), based on the formula MUAMC = MUAC − *π* × TSF.^22^ The unit used for MUAC and TSF was cm.

### Outcomes

We prospectively gathered data on patient prognosis within 2 years of discharge up to March 2020. The primary prognostic outcome was all‐cause mortality, and we investigated the cause of death (cardiovascular death or non‐cardiovascular death). After discharge, most patients were followed up in outpatient clinics at least every 3 months, according to their medical needs. For those who did not undergo in‐clinic follow‐up, prognostic data were obtained via telephone interviews regarding the clinical course at other medical facilities.

### Statistical analysis

The results for continuous variables are expressed as medians (inter‐quartile range [IQR]). Categorical variables are expressed as numbers and percentages. Patients were divided into two groups according to whether they had cachexia or not to compare patients' characteristics. The baseline patient characteristics were compared using the Mann–Whitney *U* test for continuous variables and chi‐squared test for categorical variables, as appropriate. Non‐normally distributed variables were converted to a logarithmic scale for further analysis. Multiple imputations using the ‘mice’ package in R Version 3.14.0 (The R Foundation for Statistical Computing, Vienna, Austria)^23^ were generated for 20 datasets with complemented missing values.

We calculated 100 person‐years for each participant from the day of discharge to the event occurrence or follow‐up date to evaluate incidence rates. Kaplan–Meier analysis was used to evaluate survival rates. The log‐rank test was used to evaluate the significance of the prognosis (all‐cause mortality, cardiovascular disease [CVD] mortality and non‐CVD mortality), comparing the prognosis of the patients in the cachexia or non‐cachexia groups. We further stratified the patients into two groups according to left ventricular ejection fraction (LVEF) <50% or ≥50% and performed the same analysis for all‐cause mortality by HF with reduced ejection fraction (HFrEF) and HF with preserved ejection fraction (HFpEF) groups. In addition, we evaluated the significance of all‐cause mortality, comparing the prognosis of the patients in the weight loss or non‐weight loss groups using the Kaplan–Meier analysis and the log‐rank test.

Cox regression analysis was performed to calculate hazard ratios (HRs) and 95% confidence intervals (CIs) to assess the prognostic value of having cachexia. Similarly, we also calculated HRs and 95% CIs to assess the prognostic value for all‐cause mortality of each component of Evans's criteria (weight loss, decreased muscle strength, a low fat‐free mass index, abnormal biochemistry, anorexia and fatigue). An adjusted Cox regression model was used for the Meta‐analysis Global Group in Chronic Heart Failure (MAGGIC) risk score and log‐transformed BNP.

All analyses were conducted using RStudio statistical software Version 4.2.0. *P* values <0.05 were considered statistically significant.

## Results

The FRAGILE‐HF study prospectively enrolled 1332 hospitalized patients aged ≥65 years during the study period. A total of 26 patients were excluded as cachexia could not be evaluated and as missing data based on the diagnostic criteria for cachexia. A total of 1306 patients were analysed (*Figure* *S1*). The overall median age was 81 (IQR: 74–86) years, and 57.0% were men. Following Evans's criteria, the diagnostic criteria for cachexia showed that 463 (35.5%) patients had cachexia. In addition, 778 (59.6%), 934 (73.2%), 195 (15.6%), 926 (71.0%), 564 (44.9%) and 809 (64.6%) had weight loss, decreased muscle strength, a low fat‐free mass index, abnormal biochemistry, anorexia and fatigue, respectively. Baseline patient characteristics stratified by cachexia are summarized in *Table* *1*. Patients with cachexia were older; had lower BMIs, weight at baseline and 1 year prior, and rates of comorbidities, such as dyslipidaemia, diabetes and atrial fibrillation; and were less likely to be treated with angiotensin‐converting enzyme (ACE) inhibitors, angiotensin receptor blockers (ARBs) or anticoagulants. Regarding biomarkers, cachexia was associated with higher CRP, blood urea nitrogen and BNP and lower albumin, haemoglobin and haematocrit levels. Differences in phenotypes, according to LVEF, showed that 729 (56.4%) and 563 (43.6%) patients had HFrEF and HFpEF, respectively. HF phenotypes were not associated with cachexia.

**Table 1:** Patient characteristics

	Missing data, *n* (%)	Overall	Non‐cachexia	Cachexia	*P* value
		*n* = 1306	*n* = 843; 64.5%	*n* = 463; 35.5%	
Age (years)	0	81 [74–86]	80 [74–86]	82 [76–86]	0.028
Male, *n* (%)	0	745 (57.0)	490 (58.1)	255 (55.1)	0.287
BMI (kg/m^2^)	5 (0.4)	20.9 [19.0–23.5]	21.8 [19.9–24.2]	19.2 [17.4–21.7]	<0.001
Low BMI (<18.5 kg/m^2^), *n* (%)	5 (0.4)	277 (21.2)	77 (9.1)	200 (43.2)	<0.001
Weight at baseline (kg)	0	52.2 [44.2–59.2]	54.6 [47.1–61.2]	47.1 [39.5–54.5]	<0.001
Weight at 1 year prior (kg)	215 (16.5)	57.0 [49.6–65.0]	58.0 [51.0–65.0]	55.0 [46.2–63.0]	<0.001
Weight loss rate over 1 year (%)	215 (16.5)	6.8 [2.4–11.8]	4.0 [0.9–8.3]	10.8 [7.2–15.2]	<0.001
Heart rate (b.p.m.)	0	70 [60–80]	69 [60–78]	70 [61–80]	0.039
Systolic blood pressure (mmHg)	0	112 [102–125]	112 [102–126]	112 [100–122]	0.040
Diastolic blood pressure (mmHg)	0	61 [56–68]	62 [56–69]	60 [54–68]	0.060
NYHA Class III/IV, *n* (%)	0	193 (14.8)	121 (14.4)	72 (15.6)	0.560
LVEF (%)	14 (1.1)	45.0 [32.0–60.2]	45.0 [32.2–60.0]	45.0 [31.5–61.0]	0.900
Heart failure phenotypes, *n* (%)	14 (1.1)				
HFrEF (LVEF < 50%)		729 (56.4)	466 (55.9)	263 (57.3)	0.638
HFpEF (LVEF ≥ 50%)		563 (43.6)	367 (44.1)	196 (42.7)	0.638
Comorbidities					
Hypertension, *n* (%)	0	927 (71.0)	612 (72.6)	315 (68.0)	0.082
Dyslipidaemia, *n* (%)	0	469 (35.9)	324 (38.4)	145 (31.3)	0.010
Diabetes mellitus, *n* (%)	0	470 (36.0)	326 (38.7)	144 (31.1)	0.006
Coronary artery disease, *n* (%)	0	463 (35.5)	299 (35.5)	164 (35.4)	0.986
Atrial fibrillation, *n* (%)	0	583 (44.6)	399 (47.3)	184 (39.7)	0.008
Current smoker, *n* (%)	5 (0.4)	170 (13.1)	105 (12.5)	65 (14.1)	0.426
Medications					
Beta‐blocker, *n* (%)	0	954 (73.0)	619 (73.4)	335 (72.4)	0.676
ACE inhibitor or ARB, *n* (%)	0	882 (67.5)	590 (70.0)	292 (63.1)	0.011
Loop diuretics, *n* (%)	0	1140 (87.3)	725 (86.0)	415 (89.6)	0.060
MRA, *n* (%)	0	648 (49.6)	416 (49.3)	232 (50.1)	0.793
Anticoagulants, *n* (%)	0	718 (55.0)	482 (57.2)	236 (51.0)	0.031
Laboratory data at discharge					
CRP (mg/dL)	23 (1.8)	0.27 [0.11–0.77]	0.24 [0.10–0.69]	0.34 [0.12–0.91]	0.002
eGFR (mL/min/1.73 m^2^)	2 (0.2)	52.6 [35.4–69.8]	53.4 [36.1–69.8]	51.6 [33.8–69.8]	0.461
BUN (mg/dL)	2 (0.2)	26.0 [19.8–36.0]	25.3 [19.0–35.0]	27.1 [20.0–37.8]	0.045
Sodium (mEq/L)	2 (0.2)	139 [137–141]	139 [137–142]	139 [137–141]	0.001
BNP (pg/mL)	166 (12.7)	276.1 [136.2–500.8]	248.8 [124.3–451.4]	328.8 [166.7–634.6]	<0.001
Albumin (g/dL)	36 (2.8)	3.5 [3.1–3.8]	3.5 [3.2–3.8]	3.3 [3.1–3.6]	<0.001
Haemoglobin (g/dL)	2 (0.2)	11.7 [10.3–13.1]	12.0 [10.5–13.4]	11.2 [10.1–12.6]	<0.001
Haematocrit (%)	2 (0.2)	35.9 [32.1–40.1]	36.6 [32.5–40.9]	34.6 [31.5–38.7]	<0.001
Handgrip strength (kg)	30 (2.3)	19.5 [14.1–25.0]	21.0 [15.2–27.8]	16.7 [12.5–21.9]	<0.001
Mid‐upper arm circumference (cm)	36 (2.8)	24.0 [21.6–26.0]	24.5 [22.8–27.0]	22.5 [20.1–24.5]	<0.001
Triceps skinfold thickness (cm)	54 (4.1)	1.0 [0.7–1.6]	1.0 [0.8–1.6]	0.9 [0.6–1.4]	<0.001
Mid‐upper arm muscle circumference (cm)	55 (4.2)	20.4 [18.2–22.3]	20.9 [19.1–22.8]	19.0 [16.9–21.1]	<0.001
MAGGIC risk score (points)	25 (1.9)	26 [23–30]	26 [22–29]	27 [24–31]	<0.001
All‐cause mortality, *n* (%)	21 (1.6)	270 (21.0)	145 (17.4)	125 (27.6)	<0.001
Cachexia diagnosis					
Weight loss, *n* (%)	0	778 (59.6)	315 (37.4)	463 (100)	<0.001
Decreased muscle strength, *n* (%)	30 (2.3)	934 (73.2)	512 (62.8)	422 (91.5)	<0.001
Low fat‐free mass index, *n* (%)	55 (4.2)	195 (15.6)	68 (8.5)	127 (28.3)	<0.001
Abnormal biochemistry, *n* (%)	2 (0.2)	926 (71.0)	529 (62.9)	397 (85.7)	<0.001
Anorexia, *n* (%)	50 (3.8)	564 (44.9)	248 (31.0)	316 (69.1)	<0.001
Fatigue, *n* (%)	54 (4.1)	809 (64.6)	429 (54.0)	380 (83.2)	<0.001

*Note*: Data are presented as median [inter‐quartile range] or *n* (%). Abbreviations: ACE, angiotensin‐converting enzyme; ARB, angiotensin receptor blocker; BMI, body mass index; BNP, B‐type natriuretic peptide; BUN, blood urea nitrogen; CRP, C‐reactive protein; eGFR, estimated glomerular filtration rate; HFpEF, heart failure with preserved ejection fraction; HFrEF, heart failure with reduced ejection fraction; LVEF, left ventricular ejection fraction; MAGGIC, Meta‐analysis Global Group in Chronic Heart Failure; MRA, mineralocorticoid receptor antagonist; NYHA, New York Heart Association.

**Figure 1:**
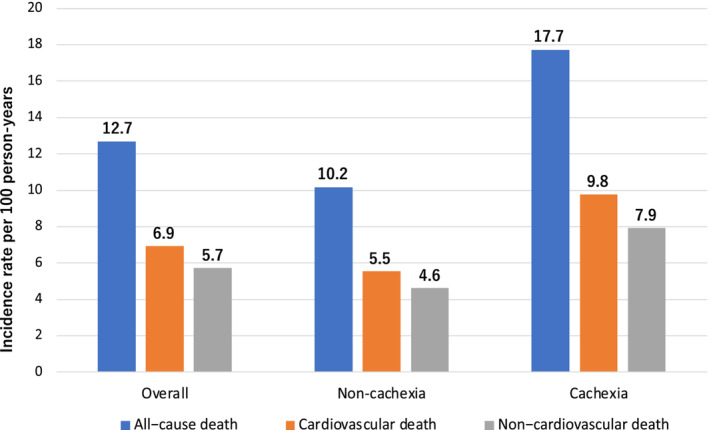
Incidence rates of all‐cause death, cardiovascular death and non‐cardiovascular death in patients in the cachexia and non‐cachexia groups.

**Figure 2:**
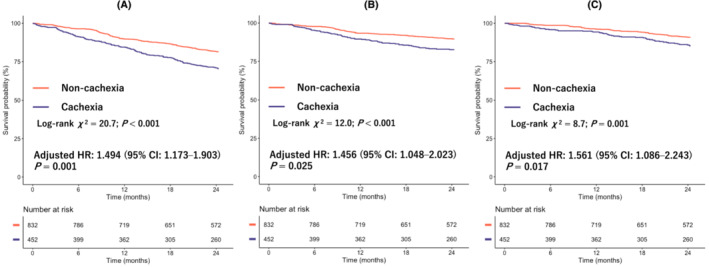
Kaplan–Meier survival curves for (A) all‐cause mortality, (B) cardiovascular disease (CVD) mortality and (C) non‐CVD mortality in patients in the cachexia and non‐cachexia groups. Hazard ratios (HRs) are adjusted with the Meta‐analysis Global Group in Chronic Heart Failure (MAGGIC) score and log B‐type natriuretic peptide (BNP). CI, confidence interval.

After excluding 22 patients with no follow‐up data, prognostic data were available for 1284 patients. A complete follow‐up of 2 years was performed in 78.0% of the total cohort (median follow‐up days, 730 days; IQR, 525–730 days), and 270 (21.0%) mortality events were observed. Cardiovascular and non‐cardiovascular deaths occurred in 148 patients (11.3%) and 122 patients (9.3%), respectively. The incident rates of all‐cause, CVD and non‐CVD events were 12.7/100, 6.9/100 and 5.7/100 person‐years, respectively (*Figure* *1*). The cachexia group had higher incident rates of all‐cause, CVD and non‐CVD events than the non‐cachexia group. Kaplan–Meier curve analysis showed that the group with cachexia had a higher event rate for all‐cause (log‐rank *χ* ^2^ = 20.7; *P* < 0.001), CVD (log‐rank *χ* ^2^ = 12.0; *P* < 0.001) and non‐CVD mortality (log‐rank *χ* ^2^ = 8.7; *P* = 0.001) than the group without cachexia (*Figure* *2*). The results of the multivariate Cox regression analyses for all‐cause, CVD and non‐CVD mortality showed that the HR of cachexia adjusted for the MAGGIC score and log BNP was 1.494 (95% CI, 1.173–1.903; *P* = 0.001), 1.456 (95% CI, 1.048–2.023; *P* = 0.025) and 1.561 (95% CI, 1.086–2.243; *P* = 0.017), respectively, compared to the non‐cachexia group.

**Figure 3:**
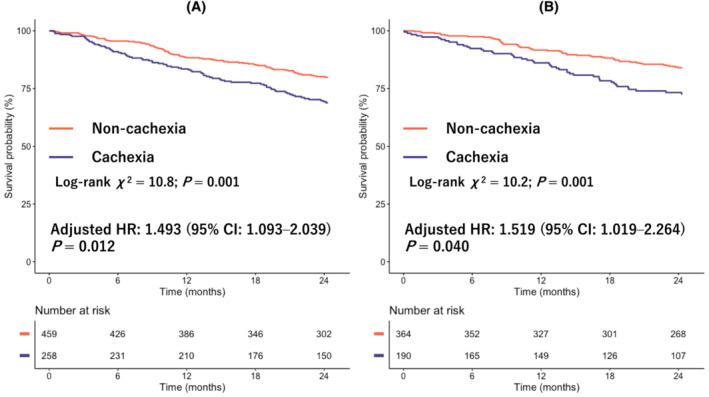
Kaplan–Meier curves for all‐cause mortality across left ventricular ejection fraction (LVEF) categories according to the presence/absence of cachexia: (A) heart failure with reduced ejection fraction (HFrEF) (LVEF < 50%) and (B) heart failure with preserved ejection fraction (HFpEF) (LVEF ≥ 50%). Hazard ratios (HRs) are adjusted with the Meta‐analysis Global Group in Chronic Heart Failure (MAGGIC) score and log B‐type natriuretic peptide (BNP). CI, confidence interval.

*Figure* *3* shows the results of Kaplan–Meier curves, log‐rank tests and multivariate Cox regression analyses for all‐cause mortality across LVEF categories according to the presence/absence of cachexia. Kaplan–Meier curve analysis showed that the group with cachexia had a higher event rate for all‐cause mortality between patients with and without cachexia in both HFrEF (log‐rank *χ* ^2^ = 10.8; *P* = 0.001) and HFpEF (log‐rank *χ* ^2^ = 10.2; *P* = 0.001). The association between cachexia stratified by the HF phenotype (HFrEF and HFpEF) and a high 2‐year mortality rate was retained even after adjusting the MAGGIC risk score and BNP (adjusted HRs and 95% CIs of the presence of cachexia: 1.493 [1.093–2.039], *P* = 0.012, in HFrEF and 1.519 [1.019–2.264], *P* = 0.040, in HFpEF).

We divided the patients into two groups based on each component of the cachexia diagnosis criteria to compare for all‐cause mortality (*Table* *2*). The HRs and 95% CIs adjusting for MAGGIC and log BNP of weight loss, decreased muscle strength, a low fat‐free mass index, abnormal biochemistry, anorexia and fatigue were 1.147 (95% CI, 0.895–1.471; *P* = 0.277), 1.514 (95% CI, 1.095–2.093; *P* = 0.012), 1.424 (95% CI, 1.052–1.926; *P* = 0.022), 1.320 (95% CI, 0.977–1.783; *P* = 0.070), 1.195 (95% CI, 0.930–1.535; *P* = 0.163) and 1.015 (95% CI, 0.782–1.317; *P* = 0.913), respectively. There was no statistically significant association between weight loss and all‐cause mortality (*Figure* *S2*).

**Table 2:** Adjusted hazard ratios of components of diagnostic criteria for all‐cause mortality

		Incident rates (per 100 person‐years)	Adjusted^a^ HR	95% CI	*P* value
Weight loss	No	11.6	1.000	Reference	
	Yes	13.4	1.147	0.895–1.471	0.277
Decreased muscle strength	No	7.3	1.000	Reference	
	Yes	14.7	1.514	1.095–2.093	0.012
Low fat‐free mass index	No	11.3	1.000	Reference	
	Yes	19.6	1.424	1.052–1.926	0.022
Abnormal biochemistry	No	8.5	1.000	Reference	
	Yes	14.5	1.320	0.977–1.783	0.070
Anorexia	No	10.9	1.000	Reference	
	Yes	14.6	1.195	0.930–1.535	0.163
Fatigue	No	12.1	1.000	Reference	
	Yes	12.7	1.015	0.782–1.317	0.913

Abbreviations: CI, confidence interval; HR, hazard ratio.

^a^ Adjusted variables for all‐cause death were the Meta‐analysis Global Group in Chronic Heart Failure score and log‐transformed B‐type natriuretic peptide.

## Discussion

This is the first prospective study on the usefulness of comprehensive cachexia assessment, as defined by Evans et al.^5^ It assessed the prognosis and the association between cachexia and life expectancy in older hospitalized adults with HF. The main findings of this study are as follows: (1) Approximately one third of older patients with HF have cachexia; (2) cachexia diagnosed by the criteria defined by Evans et al. helped predict all‐cause mortality, CVD mortality and non‐CVD mortality, while weight loss was not associated with all‐cause mortality; and (3) the presence of cachexia was associated with a poorer prognosis in both patients with HFrEF and HFpEF. The prevalence of each parameter from the overall cachexia assessment in the impact assessment of cachexia enhances prognostication, including the risk estimated by known risk factors. These findings underscore the importance of performing a more comprehensive assessment in older patients with HF using a multi‐domain cachexia assessment, as defined by Evans et al.

Many studies have evaluated cachexia in older patients with HF; however, the method of evaluating cachexia was inconsistent.^6^, ^7^, ^10^, ^11^, ^24^ In addition, most recent reports have evaluated cachexia by weight loss and biochemical abnormalities alone.^10^, ^11^, ^12^, ^25^ However, several reports have shown that cachexia assessed by weight loss in patients with HF varies widely in prevalence, ranging from 8% to 42%.^13^, ^19^ This disparity may be due to the pathogenesis of HF and the effect of treatment on weight. This study defined cachexia using Evans's criteria,^5^ which comprehensively assesses weight loss, lower muscle strength, muscle mass loss, biochemical abnormalities, anorexia and fatigue. Results showed that 35.5% of older adults with HF had cachexia; this was higher than that reported in other studies that assessed cachexia using Evans's criteria (23.8%).^6^ This may be because that study included^6^ older adults with HF who were relatively younger than the patients in our study. The study had several limitations, including having a less complete assessment of muscle strength and lean body mass, where pertinent information was often found missing. Moreover, the study included patients with stable chronic HF. Patients with HF are prone to muscle weakness and loss of lean body mass,^26^ which are strongly associated with prognosis.^3^, ^10^, ^27^ Furthermore, cachexia is more common in hospitalized patients with HF and patients with advanced symptoms than in stable outpatients with HF.^4^, ^19^, ^28^

A key finding of this study is that weight loss alone in older adults with HF was not associated with a poor prognosis. However, cachexia from Evans's criteria was associated with high mortality. It underscores the importance of performing a comprehensive cachexia assessment with multiple components in daily clinical practice.

Cachexia is defined by weight loss, which is not simply a loss of fluid or fat tissue but a state of weight loss in which changes in nutritional intake alone cannot reverse.^3^ The main characteristic of cachexia is an imbalance between the catabolic and anabolic effects of skeletal muscles. Most critical functional tissue lost by patients with cachexia is skeletal muscle, and the high levels of inflammation characteristic of patients with cachexia may activate the ubiquitin–proteasome system and cause more skeletal muscle loss.^3^ Many patients with HF also develop oedema of the gastrointestinal tract due to sodium and water reabsorption. In addition, malnutrition in patients with HF has been attributed to microcirculatory disturbances in the intestine.^29^ Intestinal ischaemia causes intestinal epithelial cell dysfunction, oedema and thickening of the intestinal wall.^29^ Moreover, malnutrition in patients with HF is associated with decreased appetite‐regulating hormones (cholecystokinin or ghrelin).^4^, ^30^ These changes are associated with anorexia in patients with HF and primarily cause protein malnutrition, resulting in muscle mass wasting. This may lead to the development of cardiac cachexia.

In this study, existing heart disease risk factors (dyslipidaemia and diabetes) were more prevalent in the non‐cachexia group. Recent studies have reported that the metabolic functional status differs between the non‐cachexia and cachexia groups due to conversion.^31^ Furthermore, due to the obesity paradox, the lipid status may improve the prognosis of cachexia.^4^, ^32^ Thus, Evans's criteria may be helpful in the early detection of cachexia because many mechanisms, needing evaluation from multiple perspectives, can cause cachexia in older adults with HF.

Weight loss is masked because most patients with HF experience congestion. However, most have cachexia and require accurate diagnoses. For this purpose, daily clinical decisions should be based on a combined assessment of muscle mass, muscle strength, inflammatory status and nutritional status, rather than weight loss alone. In addition, muscle mass assessments using dual‐energy X‐ray absorptiometry and bioelectrical impedance methods are costly. Therefore, they are not suitable for daily clinical practice. In this study, we assessed lean mass using MUAMC. MUAMC and MUAC are determinants of malnutrition and life prognosis in patients with HF and can be easily measured.^33^, ^34^, ^35^ Moreover, these measurement methods can also eliminate the effects of oedema, which is common in patients with HF.^33^ Notably, lean mass index using MUAMC was independently associated with poor prognosis in our study. The assessment used by Evans et al. is more inclusive in that it can be used despite such limitations, including oedema, and it can be used for a comprehensive evaluation. It may be helpful for the risk stratification of patients and may inform treatment and contemplated strategies for intervention.

The present study had several limitations. First, we only assessed cachexia once prior to discharge. Therefore, it is unclear how changes in cachexia conditions affect prognosis. Second, we assessed weight loss, fatigue and anorexia using a questionnaire. However, we did not exclude patients with cognitive impairments, which may have affected the results. Third, some patients had to be excluded for several reasons, including missed cachexia and prognosis assessments. This exclusion may have affected the results of this study. Finally, although this study only included older adults with HF in Japan, cachexia can also occur in younger people. Therefore, further studies are needed to determine whether the results obtained in this study can be applied to younger patients or those in Western countries.

In conclusion, this study aimed to determine the relationship between cachexia at hospital discharge and the prognosis 2 years after hospital discharge. Our results showed that older adults with HF who were evaluated for cachexia by multi‐component had worse outcomes. Assessment of cachexia by comprehensive criteria may be helpful in stratifying risk in older patients with HF and may lead to early therapeutic intervention and enhanced rehabilitation.

## Conflict of interest statement

Dr. Kentaro Kamiya received funding from Eiken Chemical Co., Ltd. Dr. Yuya Matsue is affiliated with a department endowed by Philips Respironics, ResMed, Teijin Home Healthcare and Fukuda Denshi and received an honorarium from Novartis Japan, Bayer Japan and Otsuka Pharmaceutical Co., Ltd. Dr. Nobuyuki Kagiyama is affiliated with a department funded by Philips Healthcare, Asahi Kasei Corporation, Inter Reha Co., Ltd. and Toho Holdings Co., Ltd. based on collaborative research agreements. Dr. Takatoshi Kasai is affiliated with a department endowed by Philips Respironics, ResMed, Teijin Home Healthcare and Fukuda Denshi. The other authors declare no conflicts of interest.

## Supporting information


**Figure S1.** Patient flow diagram. A total of 1306 patients were included in the primary analysis.

**Figure S2.** Kaplan–Meier survival curves for all‐cause mortality in patients in the weight loss and non‐weight loss groups.

**Table S1.** Evans criteria for diagnostic of cachexia.
