# Supplementary material for: Prognostic impact of cachexia by multi‐assessment in older adults with heart failure: FRAGILE‐HF cohort study
Source: J Cachexia Sarcopenia Muscle. 2023 Jul 11;14(5):2143–51. doi: 10.1002/jcsm.13291 (PMC10570094; doi:10.1002/jcsm.13291)
Supplement: Supplementary file 1 — Figure S1. Patient flow diagram. A total of 1306 patients were included in the primary analysis. [file JCSM-14-2143-s001.pdf]

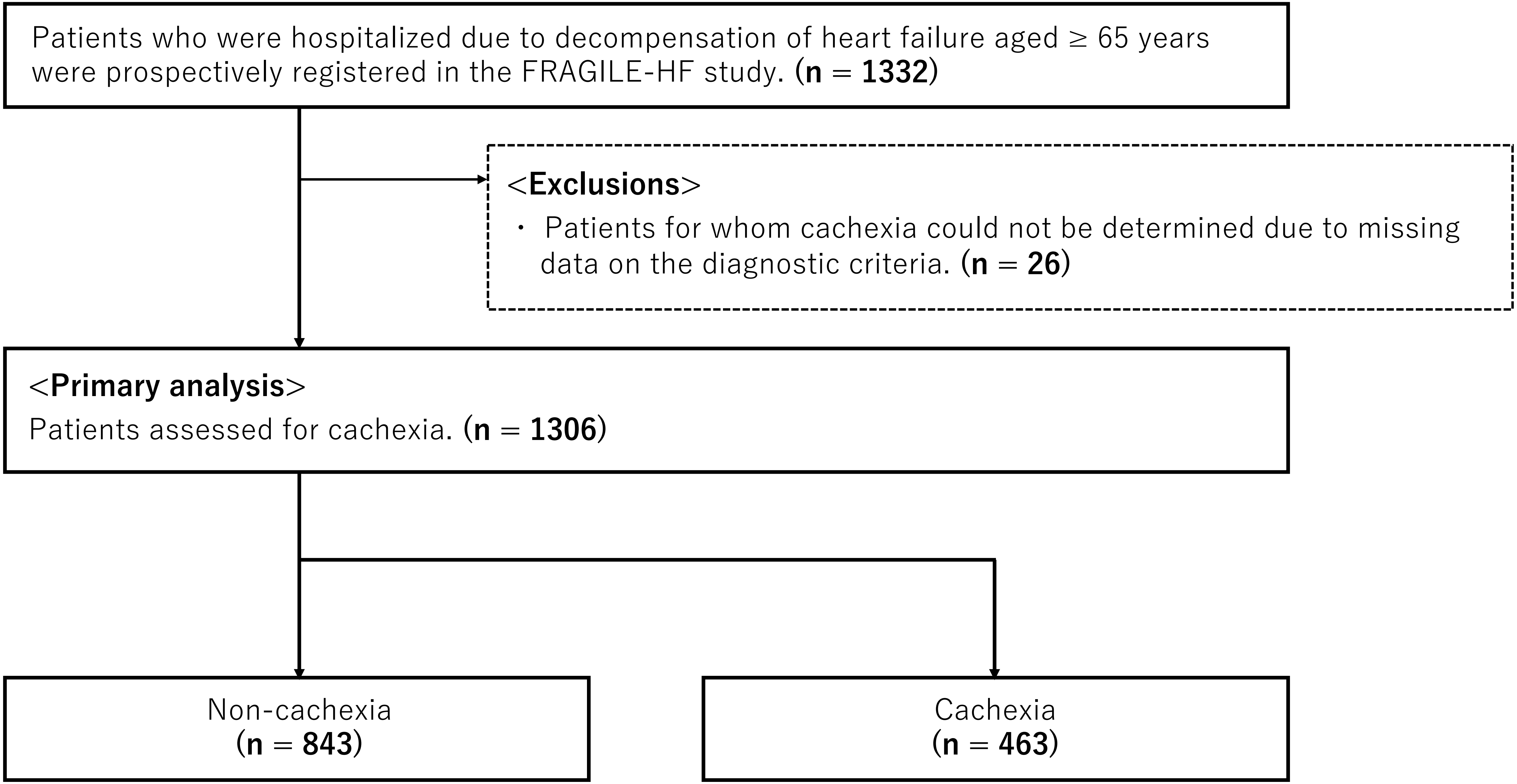
Patients who were hospitalized due to decompensation of heart failure aged ≥ 65 years were prospectively registered in the FRAGILE-HF study. (**n = 1332**)
<**Exclusions**>
・ Patients for whom cachexia could not be determined due to missing data on the diagnostic criteria. (**n = 26**)
<**Primary analysis**>
Patients assessed for cachexia. (**n = 1306**)
Non-cachexia (**n = 843**)
Cachexia (**n = 463**)
