# Supplementary figures and images for: Prognostic impact of cachexia by multi‐assessment in older adults with heart failure: FRAGILE‐HF cohort study
Source: J Cachexia Sarcopenia Muscle. 2023 Jul 11;14(5):2143–51. doi: 10.1002/jcsm.13291 (PMC10570094; doi:10.1002/jcsm.13291)

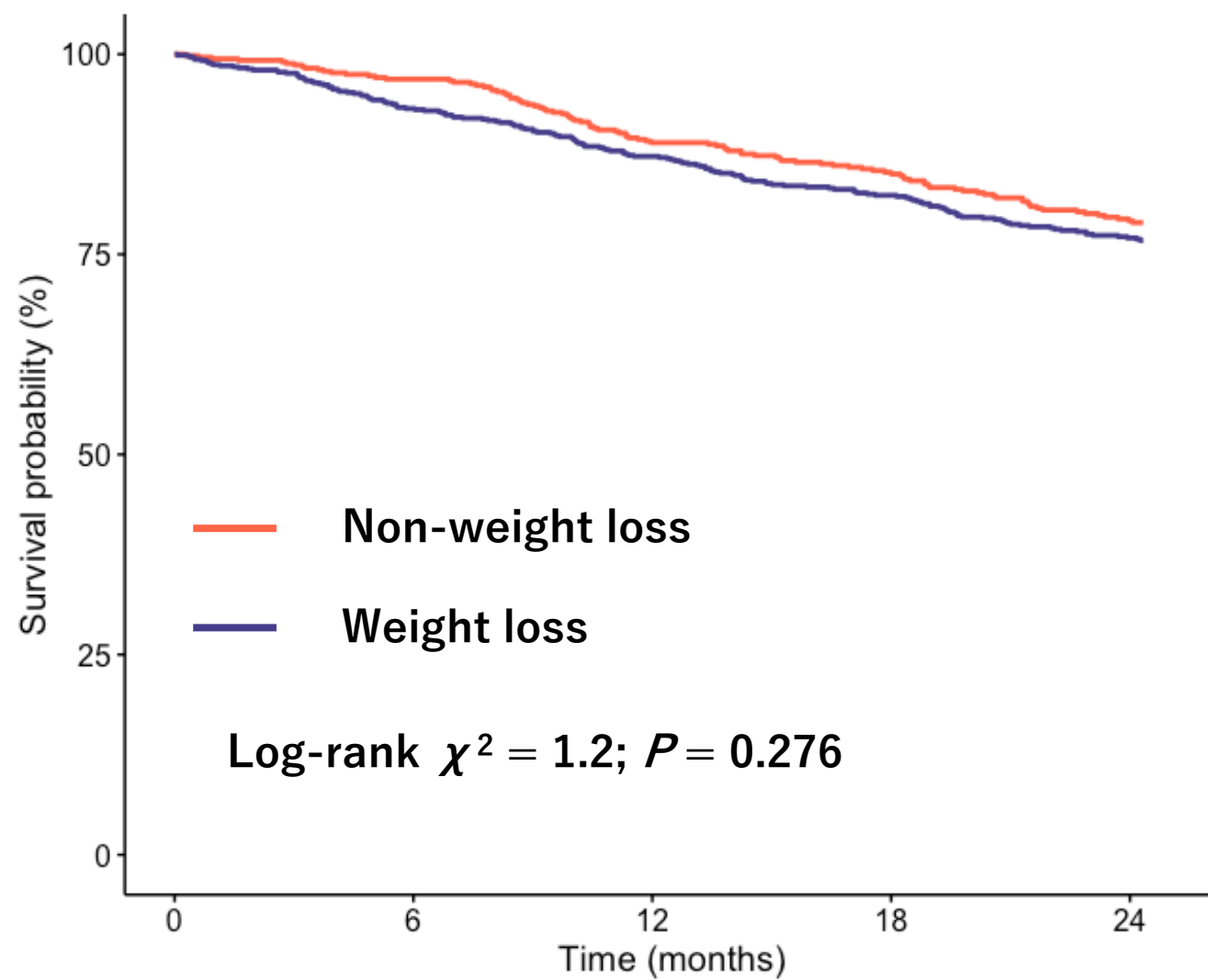

Number at risk

|     |     |     |     |     |
|-----|-----|-----|-----|-----|
| 520 | 491 | 447 | 404 | 349 |
| 764 | 694 | 634 | 552 | 483 |
| 0   | 6   | 12  | 18  | 24  |

Time (months)

Supplement: Supplementary file 2 — Figure S2. Kaplan–Meier survival curves for all‐cause mortality in patients in the weight loss and non‐weight loss groups. [file JCSM-14-2143-s002.pdf]
