# Supplementary material for: Prognostic impact of cachexia by multi‐assessment in older adults with heart failure: FRAGILE‐HF cohort study
Source: J Cachexia Sarcopenia Muscle. 2023 Jul 11;14(5):2143–51. doi: 10.1002/jcsm.13291 (PMC10570094; doi:10.1002/jcsm.13291)
Supplement: Supplementary file 3 — Table S1. Evans criteria for diagnostic of cachexia. [file JCSM-14-2143-s003.docx]

**Supplemental Table S1:** Evans criteria for diagnostic of cachexia

| Cachexia was defined by weight loss + 3 of the following criteria | |
| --- | --- |
| Criteria | Definition |
| Weight loss | At least 5% loss of weight in 1 year or body mass index < 18.5 kg/m^2^ |
| 1. Decreased muscle strength | Handgrip strength of < 28 kg for men and < 18 kg for women |
| 1. Fatigue | Physical and/or mental weariness results from little exertion (questionnaires: Yes or No) |
| 1. Anorexia | Limited food intake (< 70% of usual food intake) or poor appetite (questionnaires: Yes or No) |
| 1. Low fat-free mass index | Lean tissue depletion (mid-upper arm muscle circumference <10^th^ percentile for age and sex) |
| 1. Abnormal biochemistry | Increased C-reactive protein (> 5.0 mg/L), anemia (hemoglobin < 12 g/dL), and/or low serum albumin (< 3.2 g/dL) |
